# Supplementary material for: Bactericidal Activity of Carvacrol against Streptococcus pyogenes Involves Alteration of Membrane Fluidity and Integrity through Interaction with Membrane Phospholipids
Source: Pharmaceutics. 2022 Sep 21;14(10):1992. doi: 10.3390/pharmaceutics14101992 (PMC9607399; doi:10.3390/pharmaceutics14101992)
Supplement: Supplementary file 1 [file pharmaceutics-14-01992-s001.zip › pharmaceutics-1850974-supplementary.pdf]

pharmaceutics

MDPI

*Supplementary Material*

# Bactericidal Activity of Carvacrol against *Streptococcus pyogenes* Involves Alteration of Membrane Fluidity and Integrity through Interaction with Membrane Phospholipids

**Niluni M. Wijesundara[1,2, 3], Song F. Lee[4,7,8], Zhenyu Cheng[4], Ross Davidson[4,5,8,] David N. Langelaan[6] and H. P. Vasantha Rupasinghe[1,2,5*]**

[1]Department of Biology, Faculty of Science, Dalhousie University, Halifax, NS, Canada.
[2]Department of Plant, Food, and Environmental Sciences, Faculty of Agriculture, Dalhousie University, Truro, NS, Canada.
[3]Department of Animal Science, Faculty of Animal Science and Export Agriculture, Badulla, Sri Lanka.
[4]Department of Microbiology & Immunology, [5] Department of Pathology, [6]Department of Biochemistry & Molecular Biology, Faculty of Medicine, Dalhousie University, Halifax, NS, Canada.
[7]Department of Applied Oral Sciences, Faculty of Dentistry, Dalhousie University, Halifax, NS Canada.
[8]Canadian Center for Vaccinology, Dalhousie University, Nova Scotia Health Authority, and the Izaak Walton Killam Health Centre, Halifax, NS Canada.
[9]Department of Pathology and Laboratory Medicine, Division of Microbiology at the Queen Elizabeth II Health Sciences Centre, Nova Scotia Health Authority.
* Correspondence: author: H.P. Vasantha Rupasinghe: vrupasinghe@dal.ca; **+1 902 893 6623**

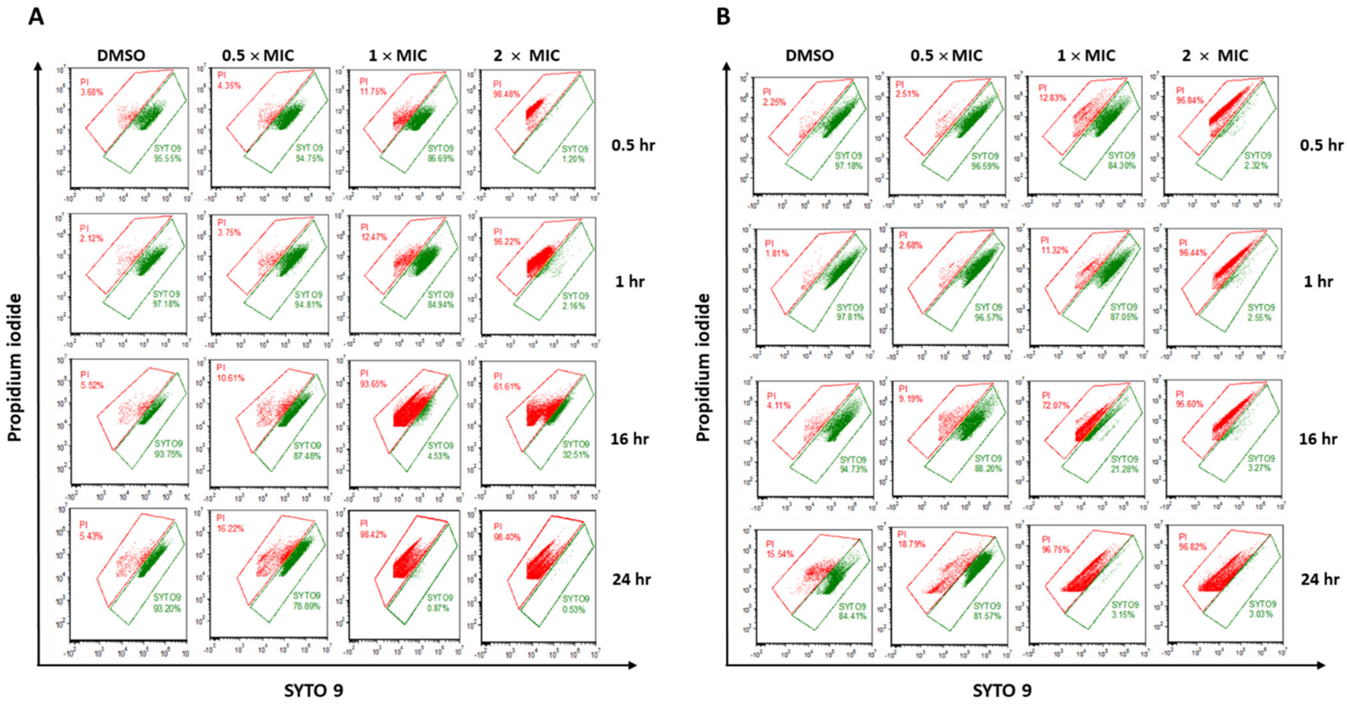


**Figure S1.** Carvacrol increases the permeability of the (A) clinical isolate and (B) spy 1558 *Streptococcus pyogenes* cell membrane in a concentration- and time-dependent manner. Bacteria cells were treated with different carvacrol concentrations or vehicle control for 0.5, 1, 16, and 24 h, stained with SYTO 9/PI and analyzed by flow cytometry. Red and green fluorescence intensity was measured using PC5.5 and FITC channels. Laser excitation/emission wavelengths of 485/542 nm for SYTO 9 and 485/610 nm for PI were used. FACS cytograms of vehicle control (0.25 % DMSO) or 0.5 × MIC, 1 × MIC, 2 × MIC of carvacrol treated spy 1558 over four different incubation periods is summarized. The population of SYTO-9 positive and PI-positive cells was shown in the green and red polygonal, respectively.

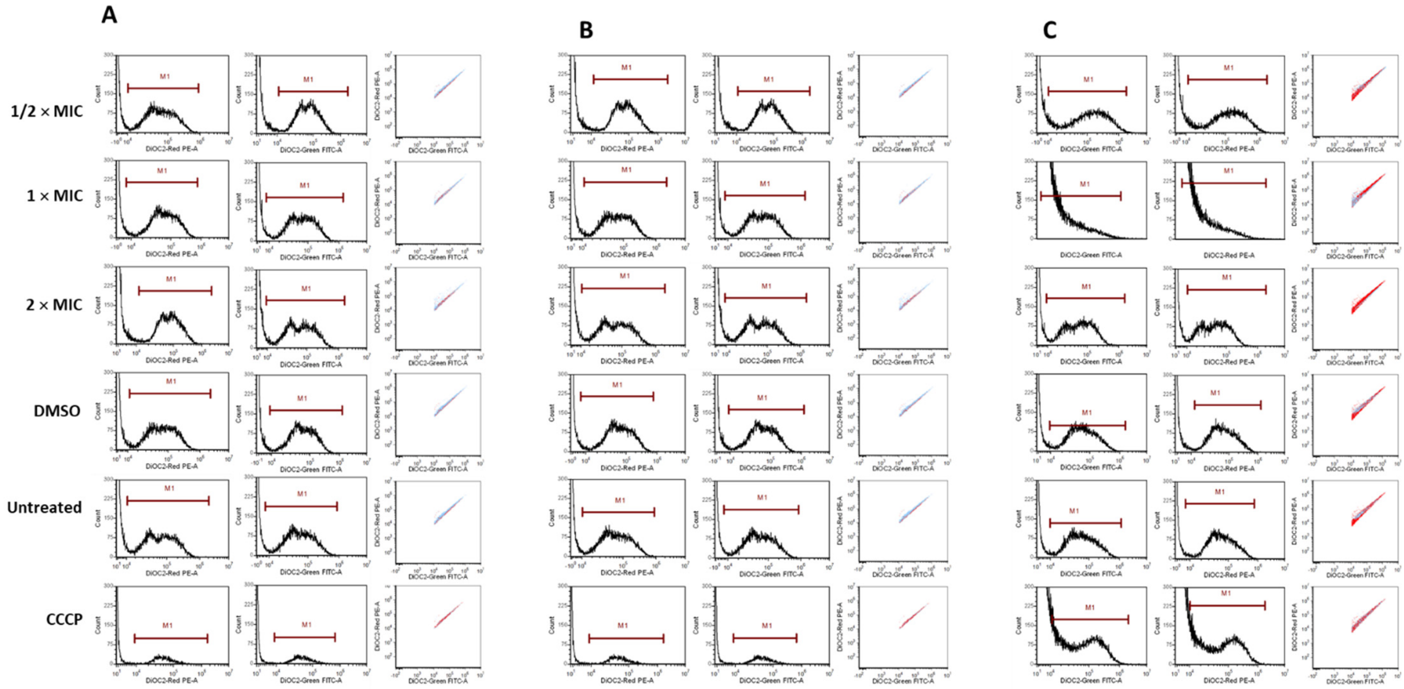


**Figure S2. Time-dependent effect of carvacrol treatments on the membrane potential.** *Streptococcus pyogenes* ATCC 19615 suspensions were treated with 2 × MIC, MIC, and 1/2 × MIC of carvacrol, or DMSO vehicle or 5 µM of CCCP, then incubated with 300 µM DiSO2 (3) for **(A)** 30 mins, **(B)** 1 h and, **(C)** 16 h MIC = 125 µg/mL. The fluorescence intensity of red and green was measured using FITC and PI channels in a FACS CytoFLEX flow cytometer. The intensity of red (left panel), green fluorescence (middle panel), and cells exhibiting both the fluorescence intensities (left panel) are shown. Red dots represent CCCP, and blue dots represent carvacrol treatments in cytometry profiles (left panel). The bar graph shows the membrane potential without (untreated control), DMSO vehicle, CCCP, or different concentrations of carvacrol treatment. Flow cytometer data were collected with log amplifications, and red: green ratios were calculated using population mean fluorescence intensities (MFI) of carvacrol treatments in either presence or absence of CCCP. Results are expressed as the ratio of MFI of red: Green ± SEM of three independent experiments. Statistical analysis was performed using one-way ANOVA, and the differences among mean were compared using Tukey's test; $^{*}P < 0.05$.

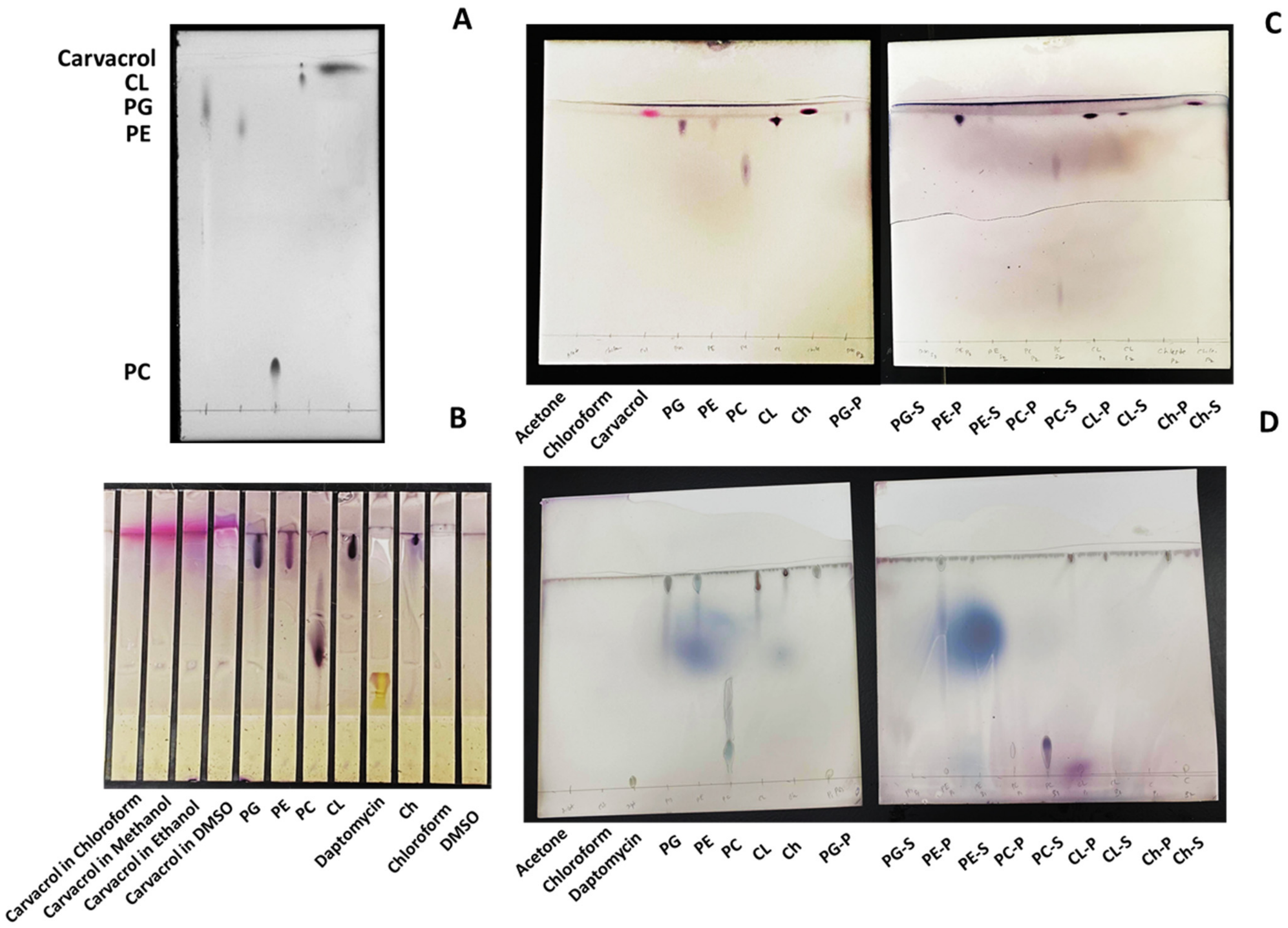


**Figure S3. Preliminary experiments of Thin-layer chromatography (TLC).** (A) UV visualization of phospholipids standards (25 mg/mL, each) and carvacrol (12.5 mg/mL) and photographs of (B) selection of better solvents and detection method for all samples, interaction with phospholipids, with (C) carvacrol and (D) daptomycin. Phospholipids: carvacrol/daptomycin (2:1 v/v, in chloroform) were incubated for 1 h, and the phospholipids were precipitated using cold acetone. Precipitant (P) and supernatant (S) were collected by high-speed centrifugation (21,000 ×g), drying, and reconstituted with chloroform. The TLC plates were developed with the solvent system of chloroform-methanol-water (65:25:10 [vol/vol/vol]). TLC plates were then stained with Alcoholic vanillin–sulphuric acid solution, heated in an oven and visualized under UV. Colors of the spots were compared and identified in photographs of TLC plates, and their mobilities (Rf) were compared with those of phospholipid standards P.G.: phosphatidylglycerol, P.E.: phosphatidylethanolamine, P.C.: phosphatidylcholine, CL: cardiolipin, Ch: cholesterol.
